# A Critical Appraisal of the Protective Activity of Polyphenolic Antioxidants against Iatrogenic Effects of Anticancer Chemotherapeutics #

**DOI:** 10.3390/antiox13010133

**Published:** 2024-01-22

**Authors:** Rosa Purgatorio, Angelina Boccarelli, Leonardo Pisani, Modesto de Candia, Marco Catto, Cosimo D. Altomare

**Affiliations:** 1 Department of Pharmacy—Pharmaceutical Sciences, University of Bari Aldo Moro, via E. Orabona 4, 70125 Bari, Italy; rosa.purgatorio@uniba.it (R.P.); leonardo.pisani@uniba.it (L.P.); modesto.decandia@uniba.it (M.d.C.); marco.catto@uniba.it (M.C.); 2 Department of Precision and Regenerative Medicine and Ionian Area, School of Medicine, University of Bari Aldo Moro, Piazza Giulio Cesare 11, 70124 Bari, Italy; angelina.boccarelli@uniba.it

**Keywords:** polyphenols, chemotherapy adjuvants, oxidative stress, cardiac toxicity

## Abstract

Polyphenolic compounds, encompassing flavonoids (e.g., quercetin, rutin, and cyanidin) and non-flavonoids (e.g., gallic acid, resveratrol, and curcumin), show several health-related beneficial effects, which include antioxidant, anti-inflammatory, hepatoprotective, antiviral, and anticarcinogenic properties, as well as the prevention of coronary heart diseases. Polyphenols have also been investigated for their counteraction against the adverse effects of common anticancer chemotherapeutics. This review evaluates the outcomes of clinical studies (and related preclinical data) over the last ten years, with a focus on the use of polyphenols in chemotherapy as auxiliary agents acting against oxidative stress toxicity induced by antitumor drugs. While further clinical studies are needed to establish adequate doses and optimal delivery systems, the improvement in polyphenols’ metabolic stability and bioavailability, through the implementation of nanotechnologies that are currently being investigated, could improve therapeutic applications of their pharmaceutical or nutraceutical preparations in tumor chemotherapy.

## 1. Introduction

Polyphenols constitute a major class of phytochemicals showing favorable effects on various pathologic conditions. They are plant-derived metabolites mainly originating from the acetate–malonate and shikimate biosynthetic pathways and they mostly exist as glycosides or are conjugated with other moieties (e.g., amines, carboxylic acids, lipids, and other phenols) [1,2]. Natural polyphenols include flavonoids and nonflavonoid compounds (e.g., phenolic acids and their esters, stilbenoids, and curcuminoids) [3].

Several studies have highlighted the relationships between dietary polyphenols and lower incidences of cancer, chronic heart diseases, and neurodegenerative syndromes [4,5,6,7]. The Mediterranean diet is associated with a reduced risk of cardiovascular disease, thanks to an adequate intake of olive oil, red wine, and anthocyanin-containing fruits and vegetables [8,9]. Other beneficial health effects, such as anti-inflammatory, antioxidant, antiallergic, antithrombotic, and antiviral activities, are related to dietary polyphenol intake [10,11,12]. Increasing lines of evidence have shown a relationship between some of the aforementioned diseases and oxidative stress resulting from the generation of reactive oxygen (ROS) and nitrogen species (RNS) [13], but no natural antioxidant has been approved so far for any therapeutic indication, except for the nutrient content claims for dietary supplements and conventional foods.

Though the clinical effectiveness of polyphenols in the prevention and treatment of cancer was recently reviewed [6,14], this article aims at evaluating the effectiveness of polyphenols as protective agents against the ROS-mediated toxic effects induced by some commonly used chemotherapeutic agents, providing a critical look at a sample of preclinical and clinical studies from the past decade, as well as highlighting some key issues related to their poor bioavailability and new nanoformulations that may increase the potential of these phytochemicals as adjuvants in tumor chemotherapy.

## 2. Methods

In this study, the databases covering biomedical and pharmaceutical sciences (e.g., Pubmed, SciFinder^®^ Scholar, and the ISI Web of Knowledge^SM^) were analyzed as sources of the literature. Clinical studies were retrieved from 2012 to October 2023, along with the main websites collecting data from clinical trials [15] and pharmaceutical patents [16]. The query strings combined the following terms: “polyphenols”, “flavonoids”, “cancer chemotherapy”, “side effects”, “toxicity”, and “bioavailability”, and the final selection of relevant studies was made by crossing combinations of these terms with “human studies” and/or “clinical trials”. For polyphenolic compounds which had proved clinically interesting as adjuvants against drug toxicity effects, updated preclinical and mechanistic information was retrieved from the literature databases.

## 3. Sources and Healthy Effects of Polyphenols

Based on the diversity of the chromane core and the hydroxyl substitution pattern, flavonoids can be divided into different groups [1,2,3], namely, flavones, isoflavones, flavanones, flavonols, flavanols, anthocyanins, and others, which mostly exist as glycosides in plants. Nonflavonoid compounds include phenolic acids (e.g., ellagic acid, protocatechuic, vanillic and gallic acids, and cinnamic acid) and their ester derivatives, stilbenoids, and curcuminoids. More complex nonflavonoid molecules are represented by stilbene oligomers, tannins, and lignins.

Besides grape, olive, blueberry, citrus fruits, broccoli, and many other vegetables and fruits [17,18,19,20], tea is a major source of dietary polyphenols [21,22]. As a frequently used beverage, its three major forms are green (unfermented), black (fully fermented), and oolong (semifermented). Catechines, in particular, epigallocatechin gallate (EGCG), are the main polyphenolic components of green tea, whereas theaflavins and thearubigins prevail in black tea. Many factors, including environmental conditions, storage, and food processing, have different influences on the content and the profile of polyphenolic components. Indeed, sun exposure, rainfall, different types of culture, and the degree of ripeness could affect their concentration and chemical diversity, as well as the aglycone/glycoside ratio [23].

**Figure 1:**
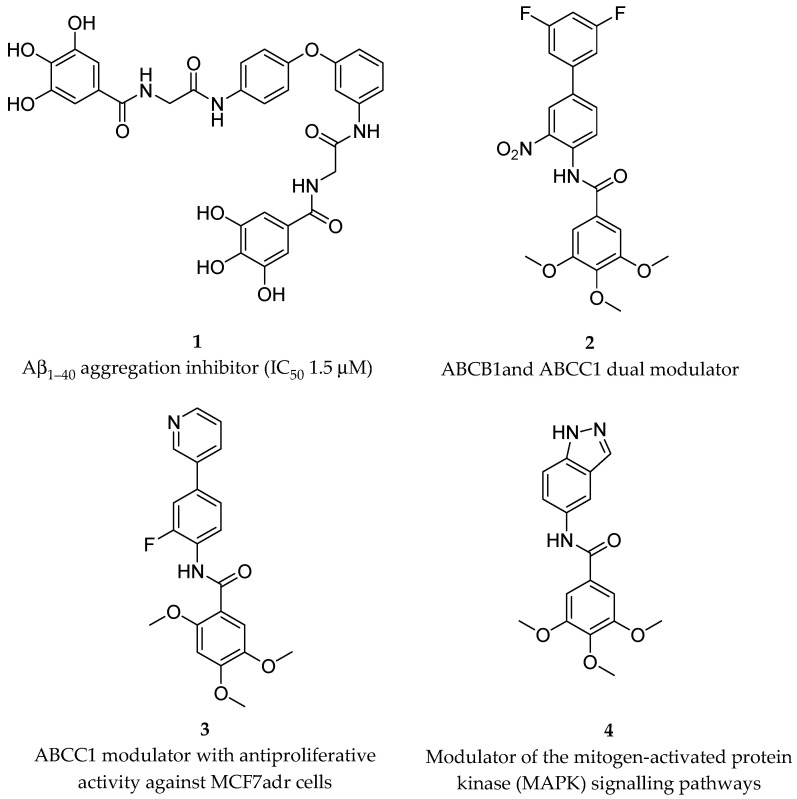
Structures and main in vitro biological data of some reported galloyl-inspired amides.

Several studies have highlighted the correlation between the consumption of polyphenol-rich foods and a lower incidence of different types of cancer, chronic heart diseases, and neurodegenerative diseases [4,5,6]. However, only a limited number of clinical studies have proven the distinct impact of dietary polyphenols on cancer prevention [24,25]. Regarding Alzheimer’s disease (AD), natural flavonoids and synthetic analogs as multitarget-directed ligands (MTDLs) have recently been reviewed [26]. Useful information on the structure–activity relationships (SARs) and pharmacophores of flavonoid-based derivatives has been reported for a number of targets playing key roles in AD’s multifactorial pathogenesis, e.g., the enzymatic inhibition of cholinesterases (ChEs), β-secretase (BACE-1), and monoamine oxidases (MAO), as well as interference with amyloid-β (Aβ) aggregation, oxidative stress, and metal imbalances. Amongst the polyphenols in clinical trials for the management of AD [27], STA-1 has entered phase 2 by Sinphar Pharmaceuticals [28] as an add-on therapy to donepezil treatment. STA-1 is an herbal remedy from traditional Chinese medicine, containing flavonoids and other polyphenolic constituents, with a broad activity spectrum [29]. Semisynthetic derivatives of gallic, protocatechuic, and vanillic acids (e.g., **1**, Figure 1) have been proven to be in vitro inhibitors of β-amyloid peptide Aβ_1–40_ aggregation [30] and potent modulators of ATP-binding cassette transporters (e.g., **2** and **3**) involved in multidrug resistance (MDR) [31,32]. Some trimethoxygalloyl-based compounds (e.g., **4**) may be able to activate TNFα-induced MAPK (mitogen-activated protein kinase) signaling in murine fibroblasts and human endothelial cells with different MAPK selectivity profiles [33].

## 4. Polyphenols and Oxidative Stress

Pathologic conditions, like cancer, cardiovascular disease, and ischemia-reperfusion injury, are related to oxidative stress caused by ROS and RNS [13]. Preventing the formation and/or scavenging of cellular ROS, such as superoxide (O_2_^−^), hydroxyl (HO), peroxyl (HOO) and alkoxyl (ROO) radicals, and RNS (e.g., peroxynitrite, ONOO^−^), is a main mechanism underlying polyphenols’ antioxidant activity [12,13,19,20]. The neuroprotective effects of kuromanin (i.e., the 3-*O*-glucoside of cyanidin) and other anthocyanins are related to their activity against nitrosative stress [34].

ROS and RNS play beneficial or deleterious roles in cells depending on their concentrations. At low concentrations, ROS and RNS modulate intracellular signaling and enzyme activity, whereas at high concentrations, they give rise to an imbalance between reactive species formation and antioxidant defenses [35,36]. Such disequilibrium leads to an increased level of the oxidant species, which can produce radical-mediated DNA injury, lipid peroxidation, and protein damage, ultimately causing cell death via apoptosis or necrosis.

**Figure 2:**
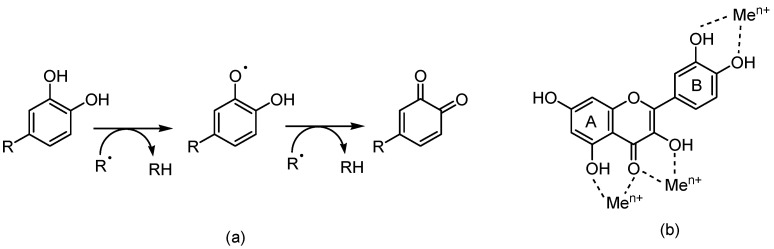
(**a**) Mechanism of free-radical (R) scavenging by the 3,4-catechol structure in the B ring of a flavonoid; (**b**) binding sites of trace metal ions (Me^n+^) to a flavanol structure (e.g., quercetin).

As reducing agents, polyphenols suppress the generation of free radicals and reduce the rate of oxidation by inhibiting the formation or deactivating the active species and precursors of free radicals. In addition to their metal (iron, copper, etc.) chelating ability, flavonoids (in particular, quercetin) inhibit ROS and RNS generation (Figure 2). Structure–antioxidant activity relationships have shown the importance of the highly conjugated aromatic ring and the hydroxylation pattern [37,38,39].

**Figure 3:**
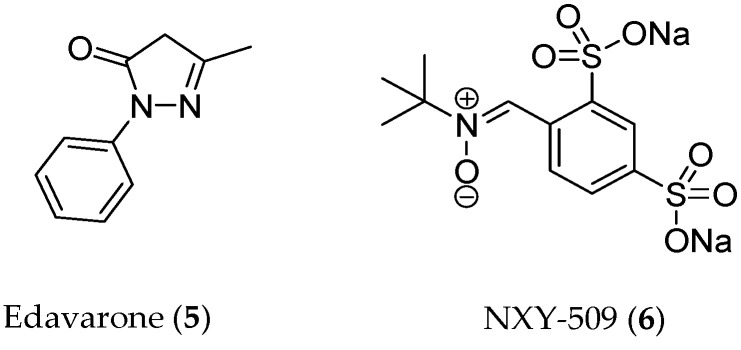
Structures of recently developed synthetic antioxidants.

However, so far, neither bioactive polyphenols nor synthetic antioxidants have been approved for any indication. To the best of our knowledge, edaravone (Mitsubishi Tanabe Pharma Corporation), a synthetic pyrazolone derivative (**5**, Figure 3) acting as a free-radical scavenger, has recently been approved for stroke and amyotrophic lateral sclerosis [40], whereas the nitrone compound NXY-059 (**6**) failed to show clinical efficacy, though it had significant effects in the preclinical treatment of acute ischemic stroke [41].

## 5. Polyphenols and Anticancer Activity

Epidemiological studies have evidenced a cause/effect correlation of cancer pathologies with urban lifestyles, diets, and environmental conditions [42,43]. Current treatments of cancer, i.e., chemo-, radio-, and immunotherapy, have variable toxic side effects for patients [44]. The search for effective, nontoxic chemotherapeutics has often turned to the natural world to discover new bioactive molecules. Among these, plant-derived polyphenols are by far the most recognized as useful dietary components with many health benefits. They are characterized by a high level of structural diversity, which in turn generates different biological properties. Epidemiological, preclinical, and clinical research has shown that the daily consumption of polyphenols is strongly correlated with the prevention of cancer. Polyphenols can exert their anticancer impact by regulating many cellular signaling pathways when acting on different target proteins [45]. Polyphenolic compounds can therefore influence carcinogenesis processes through different mechanisms; however, the main obstacles to effective treatment are high metabolic liability, weak membrane permeability, low systemic exposure, physiological fluctuation, and oxidative damage [46].

Their antitumor characteristics have been mainly ascribed to their anti-inflammatory, cell cycle arrest, antimetastatic, antiangiogenic, autophagic, antiproliferative, and apoptotic effects [43]. Polyphenols can elicit their anticancer activity by targeting cellular mechanisms, such as gene expression, cell cycle proliferation, cellular migration, and progression. The cytoprotective and anticancer properties of polyphenolic substances can generally be attributed to their antioxidant effects [47]. Polyphenols are able to (i) eliminate ROS and other free radicals; (ii) decrease DNA mutation and damage; (iii) suppress the cell cycle; (iv) induce apoptosis; and (v) down-regulate cell proliferation by means of key signaling pathway modulation (PI3K/Akt, EGFR/MAPK, NF-kB). Moreover, polyphenols may exhibit anticancer effects through different mechanisms, for example, the perforin-granzyme apoptotic pathway, mitochondria-mediated apoptosis via ROS overgeneration, and the death receptor pathway [48]. Phenolic compounds can also induce the regulation of metabolism, cell development, and the inhibition of tumor expression via the p53 pathway. They can also act by stopping DNA replication and RNA transcription, as well as by repairing the DNA damage in cancer cells [49].

## 6. Protective Effects of Polyphenols against Adverse Effects of Antitumor Therapies

Herein, several recent preclinical and clinical studies on the effectiveness of polyphenols in protecting against the adverse effects of anticancer drugs, mainly ROS-mediated toxicity, have been critically analyzed with the aim of evaluating their potential use as adjuvants in cancer chemotherapy. The pharmacological key findings are summarized in Table 1.

### 6.1. Polyphenolic Adjuvants in Anticancer Therapeutic Interventions

In a study conducted on MCF-7 cells (human breast cancer), ellagic acid was proven to (i) increase cell death, (ii) reduce cells’ capacity to form colonies, and (iii) accumulate cells in the sub-G1 (apoptotic) phase after gamma radiation treatment [50]. The effects were significantly higher for the combined treatment compared to the ellagic acid or irradiation treatment alone, thereby demonstrating the ability of ellagic acid to radio-sensitize MCF-7 cells. Interestingly, ellagic acid showed radio-protective effects on normal murine cell line in vitro.

Fractions from wine extracts, mainly containing procyanidins, catechins, and flavonols, have shown an antiproliferative effect on PC3 cells (prostate cancer) in a dose-dependent manner [51]. These fractions induced autophagy on the same cell line, thus corroborating the potential to prevent the disease.

In a recent in vitro study [52], ovarian cell lines were treated with oleuropein (a phenolic compound present in the fruits and leaves of olive trees). In particular, the authors showed that after using oleuropein to treat A2780 and A2780 cisplatin resistance cell lines, the expression of p21 and p53 increased, while the expression of Bcl-2 decreased. As a result, oleuropein was able to induce apoptosis, reduce cell proliferation, and reduce resistance to cisplatin in ovarian cell lines.

Hydroxytyrosol, the product of oleuropein hydrolysis, is an effective anti-inflammatory and antioxidant polyphenol. It is able to reduce the nephrotoxicity from cisplatin by inhibiting chemokine-like factor 1 (CKLF1) involved in inflammation pathways and to induce anti-oxidative stress and anti-apoptosis activities in the kidneys of mice [53].

**Figure 4:**
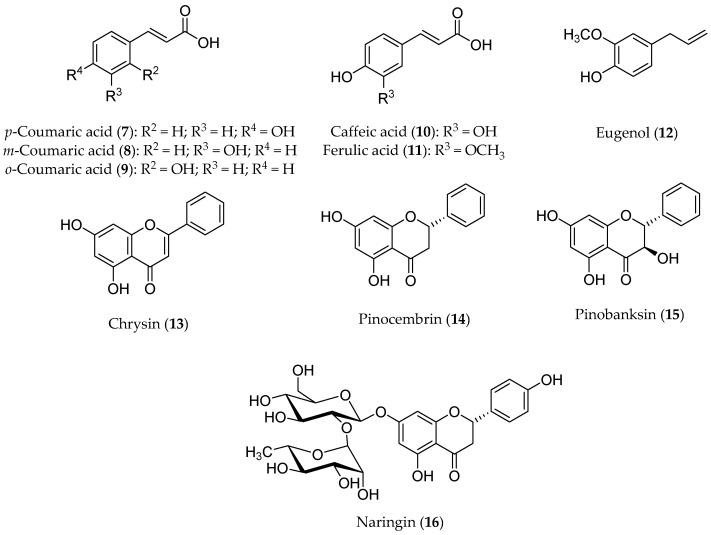
Structures of some polyphenolic components of honey.

Honey contains a mixture of different active compounds (Figure 4), including coumaric acids (**7**–**9**), caffeic acid (**10**), ferulic acid (**11**), eugenol (**12**), and flavonoids, such as quercetin, apigenin, chrysin (**13**), pinocembrin (**14**), pinobanksin (**15**), and naringin (**16**) in different percentages depending on the floral source and geographical origins. An increasing amount of evidence has attributed a potential chemopreventive activity to honey [54]. In fact, multi-floral honey prevented the formation of breast cancer induced by 7,12-dimethylbenz(a)anthracene (DMBA) in a rat model [55]. Moreover, an increased level of bone marrow lymphocytes and peritoneal macrophages in mice suggested the activation of the immune system [56]. Oral mucositis (OM), which is one of the most common side effects of chemotherapy, could be reduced by honey, thanks to its capacity to increase the immune system response [57]. This effect was confirmed by a double-blind randomized clinical trial in which the patients affected by OM after chemotherapy were treated with betamethasone, honey, and a combination of honey and coffee [58].

Patients with head and neck cancer treated with radiotherapy showed a reduction in oral side effects (xerostomia) after consuming thyme honey [59], and manuka honey and talk honey induced a reduction in liver and kidney toxicity via cisplatin in rats [60]. This organ-protective effect could be due to honey’s free-radical scavenging as well as anti-inflammatory and anti-apoptotic activities. These data suggest the protective effect of honey and its promising application.

To induce the same protective effect on oral mucosa, a clinical trial (NCT05994638, 2023) [61] is starting to recruit patients receiving a polyphenol-rich aerosol for minimizing side effects in patients after radiation therapy. A group of 10 patients with head and neck cancer who have undergone radiotherapy will orally receive an aerosol constituted of polyphenol-rich plant extracts, hyaluronic acid, *Cetraria islandica*, and vitamin B3 for one month. Another clinical trial (NCT06017661, 2023) [62] is using a standard commercial product, ‘nutridrink’, enriched with a preparation of plant extracts rich in polyphenolic compounds as support for recovering patients undergoing gastrointestinal tumor resection.

In a recent patent (CN111447940; 2020) [63], quercetin and its analogues were used to provide novel compositions and methods for the treatment of radiation-induced bystander effects (RIBEs) resulting from radiation exposure. Moreover, in a recruiting clinical study (NCT05984888, 2023) [64], some patients affected by breast cancer were treated with the MIND (Mediterranean Intervention for Neurodegenerative Delay), with the aim of protecting the brain from the toxic side effects of chemotherapy. The MIND is a diet with anti-inflammatory nutrients (e.g., omega-3 polyunsaturated fatty acids (PUFAs), carotenoids, B-vitamins, and polyphenols) which may help alleviate negative cognitive outcomes from cancer treatments. Another clinical trial (NCT02195960, 2023) [65] seeks to evaluate the effects of polyphenol-rich food supplementation against the toxic side effects of breast cancer radiotherapy.

### 6.2. Activity against ROS-Mediated Effects of Chemotherapeutics

**Figure 5:**
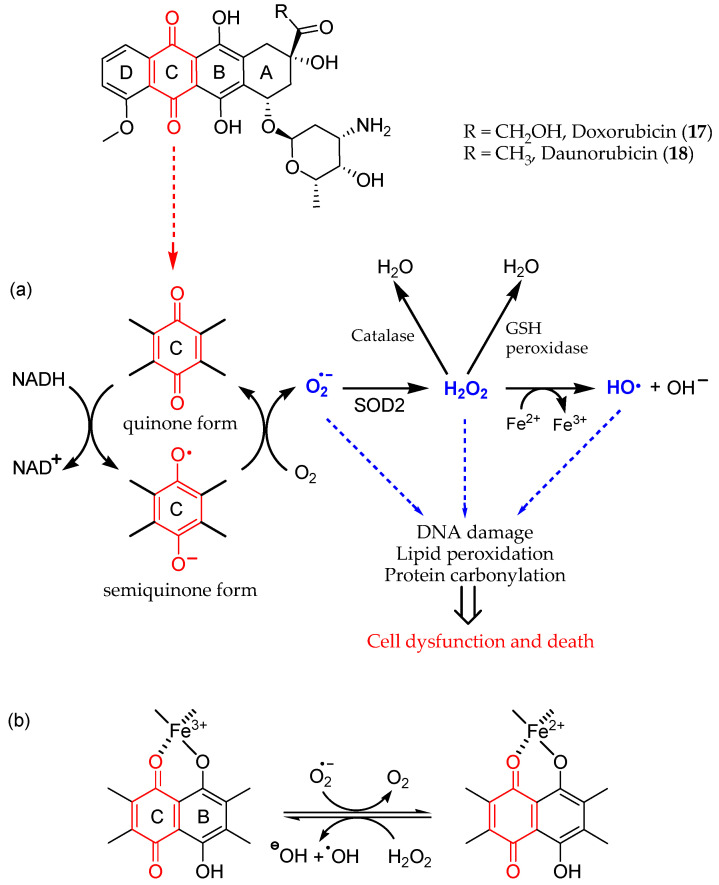
Redox cycling of anthracyclines **17** and **18**, leading to ROS production and cardiotoxicity; it involves (**a**) the quinone C ring and (**b**) iron chelation (metal ion binding sites in B and C rings).

Anthracyclines are anticancer antibiotics characterized by an anthraquinone moiety branched with an amino sugar at C-7. Doxorubicin (**17**) and daunorubicin (**18**) (Figure 5), isolated from the bacteria *Streptomyces peucetius*, were the earliest drugs of this family that entered clinical practice for cancer treatment [66]. Daunorubicin is effective in acute lymphocytic and myeloid leukemia, while doxorubicin is a component of polypharmacological protocols for treating solid tumors (e.g., breast cancer, soft tissue sarcomas, and aggressive lymphomas). Even though it is a common chemotherapeutic, the clinical use of doxorubicin is limited by its dose-dependent cardiac toxicity, which may lead to severe and irreversible forms of cardiomyopathy [67,68]. Indeed, anthracyclines can induce the early onset of progressive chronic cardiotoxicity, usually within one year of treatment [69]. Cardiomyopathy may persist or advance even after the discontinuation of therapy [70].

The mechanistic explanation for iatrogenic cardiotoxicity lies in the overproduction of ROS. Doxorubicin quinone can be reversibly reduced to semiquinone, an unstable metabolite whose futile redox cycle within mitochondria leads to ROS overload, especially the superoxide radical anion O_2_∙^−^. As shown in Figure 5, the reduction in doxorubicin quinone (C ring) to semiquinone is catalyzed by NADH-dependent enzymes. The semiquinone in turn donates a single electron to O_2_, thereby generating O_2_∙^−^ and recycling itself to quinone. Superoxide dismutase 2 (SOD2) catalyzes the transformation of O_2_∙^−^ in H_2_O_2_, which can be detoxified by catalase or glutathione (GSH) peroxidase in the presence of GSH or converted into the highly reactive hydroxyl radical (HO) in the presence of endogenous Fe^2+^ through the Fenton reaction.

The highly reactive HO^∙^ can in turn generate lipid radicals and other ROS. In addition to ROS, the RNS peroxynitrite (ONOO^−^) is generated in cardiomyocytes following doxorubicin administration, most likely due to the reaction between the O_2_∙^−^ generated from mitochondria and nitric oxide (NO) [71]. In addition, iron ions (Fe^2+^/Fe^3+^) have been shown to play a crucial role in this process. Fe^3+^ is able to react with hydrogen peroxide to yield reactive hydroperoxyl radicals (HOO) and to form chelates with the C11-C12 β-hydroxycarbonyl system of doxorubicin (Figure 5) [72]. Iron accumulates in cardiomyocytes during doxorubicin treatment, probably because of its capability to interfere with the main iron-transporting and iron-binding proteins [73]. In preclinical and clinical studies of anthracycline-induced cardiotoxicity, iron chelators, such as dexrazoxane, showed promise [74]. Other molecules, such as amifostine and mesna for example, have also been evaluated as cardioprotective auxiliary agents in preclinical studies [75].

#### 6.2.1. Preclinical Findings

**Figure 6:**
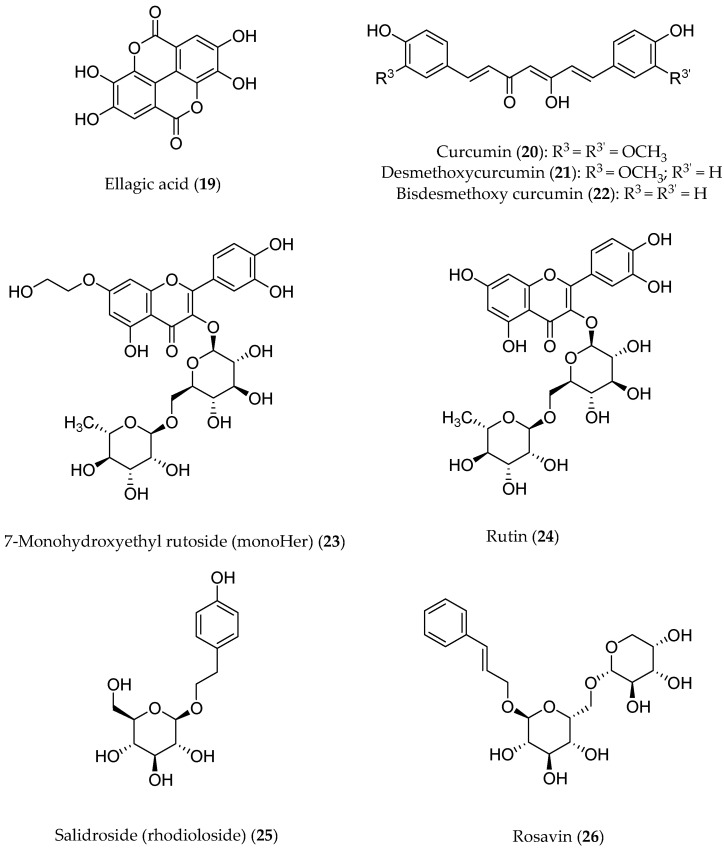
Structures of polyphenols that have been pharmacologically investigated.

Ellagic acid (**19**, Figure 6) is a product of the hydrolysis of ellagitannins. Recent pharmacological studies have demonstrated that **19** acts as a free-radical scavenger, with several health benefits, such as anti-inflammatory, antihepatotoxic, antisteatosic, anticholestatic, antifibrogenic, antidiabetic, hypolipidemic, and antiatherosclerotic effects [76,77]. Moreover, ellagic acid has been proven to inhibit type-B monoamine oxidase (MAO-B) [78], thereby protecting rat brains from 6-hydroxydopamine-induced neuroinflammation in a model of Parkinson’s disease [79] and preventing scopolamine- and diazepam-induced cognitive impairments [80]. In male Wistar rats, orally administered **19** was proven to attenuate the doxorubicin-induced oxidative process in myocardial tissue [81].

Gallic acid, a potent free-radical scavenger [82], is a product of the hydrolysis of gallotannins. Doxorubicin-treated albino rats developed severe alopecia, and their fur became scruffy [83]. A 60% mortality rate was observed in the doxorubicin group, whereas in animals treated with gallic acid orally administered at doses of 15 mg/kg and 30 mg/kg, the mortality rate decreased to 30% and 15%, respectively. Gallic acid showed effectiveness in the functional recovery of the heart, with a significant reduction in cardiac injury, which may be related to its antioxidant properties [84].

Medicinal uses of turmeric (*Curcuma longa* L., *Zingiberaceae*) arise from its content of volatile oil and curcuminoids (Figure 6, **20**–**22**) [85]. It has been reported that turmeric has anti-inflammatory, hepatoprotective, antiviral, and anticancer activities, and it might have neuroprotective effects [86,87]. Recently, the medicinal chemistry of curcumin has been reviewed in depth, and new research developments on curcuminoids have been widely discussed [88]. Extensive investigations over the past quarter century, including over a hundred clinical studies of curcuminoids against several diseases, have addressed the pharmacokinetics, safety, and efficacy of turmeric [89].

The administration of a single dose of doxorubicin to a group of male rats was compared with a group receiving doxorubicin and an alcoholic extract of *C. longa* L. via an oral gavage. Compared to the controls, the doxorubicin-treated animals showed a 50% increase in mortality. In rats co-treated with turmeric extracts, not only was the mortality rate significantly diminished, but the heart weight and heart/bodyweight ratio significantly increased. Turmeric was proven to protect animals against acute doxorubicin-induced cardiotoxicity, ameliorate cardiac enzymes, and modulate the pathways triggering cardiac apoptosis, decreased levels of GSH, and the overproduction of oxidant radicals [90].

#### 6.2.2. Evaluation of Clinical Studies

The flavonoid 7-mono-*O*-(β-hydroxyethyl)rutoside (monoHER) (**23**, Figure 6), a semi-synthetic derivative of rutin (**24**) bearing rutinose (α-l-rhamnopyranosyl-(1→6)-β-d-glucopyranose) as the disaccharide moiety, has been shown to protect mice against doxorubicin-induced cardiotoxicity without adverse effects at a very high dose (500 mg/kg) [91]. Based on these results, clinical trials were performed to evaluate its protective effects in cancer patients. MonoHER, administered intravenously at a 1500 mg/m^2^ dose 60 min before doxorubicin was administered, was evaluated through an endomyocardial biopsy, but the benefits observed in these preclinical studies were not confirmed [92]. These conflicting results might be attributable to interspecies differences in ADME (absorption, distribution, metabolism, and excretion). However, the antitumor activity of doxorubicin appeared to greatly improve, even displaying a partial remission of metastatic soft-tissue sarcoma in some patients. These results are somehow in agreement with the potentiating anti-proliferative effects observed in vitro for a number of flavonoids [93].

Another noteworthy clinically investigated polyphenol is salidroside (i.e., tyrosol glucoside, **25**) found in *Rhodiola rosea* and used in traditional Tibetan medicine. Salidroside (Figure 6), along with the less active rosavin **26** (a cinnamyl alcohol glycoside bearing α-l-arabinopyranosyl-α-d-glucopyranoside as the disaccharide moiety), was reported to play a role in reducing mitochondrial-generated ROS and apoptosis signaling [94]. Pretreatment with salidroside appears to significantly reduce in vitro both ROS and mitochondrial superoxide overproduction [95] as well as to arrest the cell cycle and apoptosis in human breast cancer cells [96].

Furthermore, **25** showed antioxidant-related cardiovascular protection [97]. These results led to clinical studies to assess its effectiveness in protecting against cardiac dysfunctions induced by epirubicin in sixty patients with histologically confirmed breast cancer. In this trial, all the patients had a scheduled cumulative epirubicin dose of 400 mg/m^2^. Although the oral co-administration of **25** and epirubicin was well tolerated in all of the patients, no significant differences in the protection from epirubicin-induced cardiotoxic effects were found compared to the placebo groups, once again suggesting that most likely the poor bioavailability of the polyphenolic phytochemicals in humans is a major factor limiting its clinical application [98].

**Table 1:** Major outcomes from pharmacological studies on the protective activity of polyphenols against toxicity effects of antitumor drugs.

	Source	Polyphenol/s	Key Findings	Ref.
In vitro models	Wine extracts	Ellagic acid	Pro-apoptotic effect after gamma irradiation on MCF-7 cells	[50]
		Procyanidins, catechins, flavonols	Antiproliferative effect in PC3 prostatic cancer cells	[51]
	Olive tree	Oleuropein	Apoptosis induction, cell proliferation reduction, and resistance to cisplatin Reduction in ovarian carcinoma cell lines (A2780)	[52]
		Salidroside	ROS and superoxide reduction in breast cancer lines	[95]
			Cell cycle arrest of breast cancer lines	[96]
Animal models	Honey	Coumaric acid, ferulic acid, caffeic acid, eugenol, flavonoids	Prevention of DMBA-induced breast cancer in rats	[55]
	Olive tree	Hydroxytyrosol	Activation of immune system in mice	[57]
			Inhibition of chemokine-like factor 1 (CKLF1), as well as anti-oxidative and anti-apoptosis effects in kidneys of mice during cisplatin treatment	[53]
		Ellagic acid	Attenuation of doxorubicin-related oxidative stress in male Wistar rats	[81]
		Gallic acid	Mortality reduction and heart functional recovery in doxorubicin-treated rats	[84]
	Turmeric alcoholic extract	Curcuminoids	Reduction in doxorubicin-treated male rats mortality as well as increase in heart weight and heart/bodyweight	[90]
		MonoHER	Protection from doxorubicin cardiotoxicity in mice	[91]
Clinical studies	Honey	Coumaric acid, ferulic acid, caffeic acid, eugenol, flavonoids	Oral mucositis reduction in double-blind clinical trial	[58]
		MonoHER	Doxorubicin activity improvement (partial metastatic soft-tissue sarcoma remission)	[93]
		Salidroside	In co-administration, no significant protection against epirubicin-related cardiotoxicity although well tolerated	[98]
	Aereosol	Polyphenols	Minimization of oral side effects in patients after radiation therapy	[61]
	Nutridrink		Recovery of patients undergoing gastrointestinal tumor resection	[62]
	MINDS		Brain protection from toxic side effects of chemotherapy	[64]
	Rich-food		Reduction in radiotherapy side effects in those with breast cancer	[65]

### 6.3. Bioavailability Issues

A main hurdle to the therapeutic application of polyphenols is their poor bioavailability. Despite their antioxidant, antiphlogistic, and anticancer pharmacological activities, which may synergistically cooperate in antitumor chemotherapy, polyphenols show poor bioavailability, which strongly limits their efficacy. Several factors, such as low solubility/permeability, photochemical isomerization, auto-oxidation, and hepatic/intestinal rapid metabolic processes, just to name the main ones, negatively affect their bioavailability and in fact represent major obstacles to their therapeutic use. Nanodelivery systems may have the potential to improve their therapeutic efficacy. While the reader may refer to recent, more exhaustive reviews on these topics [4,10], herein we would like to draw attention to some promising applications in CNS diseases [46] or in cancer prevention and therapy [99].

**Figure 7:**
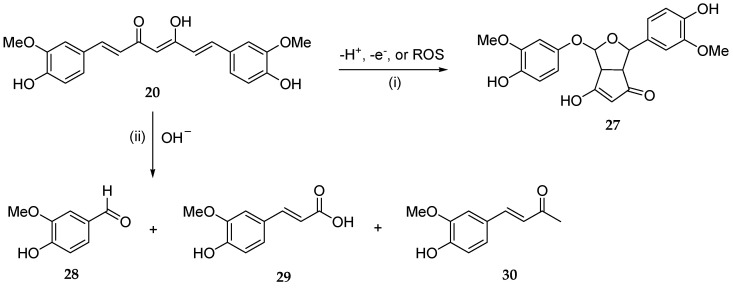
Main chemical degradation reactions of curcumin (**20**): (i) autoxidation in aqueous buffered medium; (ii) solvolysis under alkaline pH in aqueous buffer.

For example, numerous studies have proven that curcumin (**20**) is chemically and metabolically unstable and thereby poorly bioavailable [88]. A spectroscopic analysis revealed that a major degradation product (**27**) is formed by the autoxidation of **20** [100], whereas three minor degradation products, namely vanillin (**28**), ferulic acid (**29**), and the ketone product **30,** are generated via a solvolysis reaction in an aqueous alkaline buffer [101] (Figure 7). Pharmaceutical nanotechnologies may implement efficient delivery systems aimed at improving the bioavailability of polyphenols [102].

### 6.4. The Clinical Promise of Nanothecnology-Based Delivery Systems

A major advantage of the particles within a range of less than 100 nm (nanoparticles) is represented by the high surface-area-to-volume ratio [103]. In general, nanoformulations (liposomes, micelles, natural and synthetic nanoparticles, metal nanoparticles, and microspheres) may lead to improved bioavailability, biodistribution, and specificity, as well as provide the optimal pharmacokinetics for drugs delivered to tumor sites [104,105,106]. Some examples of optimized nanoformulations of plant-derived polyphenols are reported below.

The effects of polyphenolic extracts from green tea (GTE), red wine (RW) lees, and/or lemon (L) peel, alone and in combination with antitumor drugs, were investigated on the growth and development of different transplanted experimental tumors [107]. Nanosized forms of these extracts (NanoGTE, NanoGTRW, or NanoGTRWL) were produced using spray drying technology (with a 10–45 nm particle size). The total phenolic composition for the extracts ranged from 18.0 to 21.3 g/100 g for each formulation. The antitumor properties of the polyphenolic extracts and biocomposites were tested in murine-transplanted tumors, namely sarcoma 180, solid Ehrlich carcinoma, Ca755 mammary carcinoma, and B16 melanoma. The reduction indices of doxorubicin cardiotoxicity and cisplatin nephrotoxicity suggest the beneficial effects of polyphenols in green tea, red wine lees, and lemon peels as nanoextracts. This study suggested the promising development of GTE and nanoextracts as auxiliary agents in anticancer treatment.

Luteolin, one of the flavonoids of celery, green pepper, honey, and chamomile tea, showed inhibitory effects against the transcription factor Nrf2 [108]. The exposure to carcinogenic molecules activates the Nrf2 pathway, inducing the elimination of carcinogenic reactive intermediates and consequently resistance to chemotherapeutic agents. Luteolin could sensitize cancer cells to chemotherapeutic agents through the inhibition of Nfr2. Luteolin-phytosome was proven to be a potential drug delivery system able to increase the efficacy of doxorubicin in human MDA-MB 321 breast cancer cells [109]. The real-time quantitative PCR (qRT-PCR) analysis showed that phyto-luteolin suppressed the mRNA expression of Nfr2, as well as the expression of the genes of HO-1 and MDR-1 more than luteolin alone in human MDA-MB 321 breast cancer cells [110]. The cytotoxicity data showed that nanoformulations were able to inhibit the growth of MDA-MB231 cells better than luteolin or doxorubicin alone.

Another study explored the synergistic effect of resveratrol and 5-fluorouracil using PEGylated liposomes. This nanoformulation was tested in vitro on a head and neck cancer cell line (NT8e). The data showed a cytotoxicity increase in nanoformulations (liposomes) compared to the free drug [111].

The poor ADME properties of curcumin (**20**) could be overcome through nanoformulations, as recently shown by several studies. Different nanocarriers were employed, ranging from polymeric or solid lipid nanoparticles to nanocrystals, nano-emulsions, and nano liposome-encapsulated curcumin, just to mention the most important applications [112,113]. Limited to anticancer treatment, curcumin-loaded *N*-dodecyl-chitosan-HPTMA-coated liposomes showed an increased sensitivity of the mouse melanoma cell line B16F10. These tumor cells can tolerate **20** at a concentration of about 10 µM, while the cytotoxicity of the chitosan-based formulation can be observed at lower concentrations (2.5 µM) [114]. Actually, a number of studies have demonstrated that the nanoparticle encapsulation of **20** is not always beneficial. For example, curcumin-loaded chitosan/polycaprolactone nanoparticles exhibited cytotoxicity on cervical cancer and choroidal melanoma (HeLa and OCM-1 cell lines, respectively) to the same extent as free curcumin [115]. Furthermore, mPEG 2000–curcumin conjugates were equipotent to unbound curcumin against a panel of carcinoma cell lines [116].

The biodistribution and efficacy of different curcumin nanoformulations have been investigated in many in vivo studies to assess the therapeutic potential of these release systems [114]. Curcumin-loaded MPEG-PCL polymeric micelles showed a stronger antiproliferative effect in mice LL/2 pulmonary carcinoma compared to free curcumin [117]. Finally, curcumin nanoformulations were investigated in human clinical trials for many years, showing clinical benefits for patients with some solid tumors and multiple myeloma. The effects and concentrations in normal and cancerous tissues after the administration of curcumin formulations were compared; safety and immune responses were taken, in each study, as the primary and secondary outcomes, respectively [114].

## 7. Conclusions and Perspectives

The literature provides a wealth of information about numerous health-related properties, including the anticarcinogenic activity, of plant-derived polyphenols. Pharmacological data support several mechanisms for the activity of flavonoids in inhibiting cancer onset and progression. Polyphenols could play a significant role in protecting patients from the adverse effects of anticancer chemotherapeutics, especially those related to drug-induced oxidative stress. Herein, both preclinical and clinical findings regarding the effectiveness of polyphenols, or their synthetic analogs and derivatives, were reviewed with a focus on their capacity to protect against ROS-mediated toxic effects induced by antitumor chemotherapeutics. The only clinically tested flavonoid glycoside, monoHer (**23**), showed protective activity against doxorubicin-induced cardiotoxicity in mice, but its effect was not confirmed in human studies. Salidroside (**25**), which induces cell cycle arrest and apoptosis in human breast cancer cells, though tolerated in all patients, did not show significant protective effects against anthracycline-related cardiotoxic effects. This apparent dichotomy could be explained by considering several factors, such as species-related differences in metabolism and bioavailability, and issues related to dosing. Studies aimed at establishing adequate dosing and delivery systems are needed, whereas a promising research area is dedicated to the development of nanoformulations as a bioavailability booster for polyphenolic phytochemicals.

## Data Availability

No new data were created or analyzed in this study. Data sharing is not applicable to this article.
